# YOLO-ERCD: An Upgraded YOLO Framework for Efficient Road Crack Detection

**DOI:** 10.3390/s26020564

**Published:** 2026-01-14

**Authors:** Xiao Li, Ying Chu, Thorsten Chan, Wai Lun Lo, Hong Fu

**Affiliations:** 1 College of Computer Science and Software Engineering, Shenzhen University, Shenzhen 518060, China; lixiao@eduhk.hk; 2 Department of Mathematics and Information Technology, The Education University of Hong Kong, Hong Kong, China; 3 Hong Kong Productivity Council, Hong Kong, China; thorstenchan@hkpc.org; 4 Department of Computer Science, Hong Kong Chu Hai College, Hong Kong, China; wllo@chuhai.edu.hk

**Keywords:** Road Crack, YOLO, deep learning, object detection

## Abstract

Efficient and reliable road damage detection is a critical component of intelligent transportation and infrastructure control systems that rely on visual sensing technologies. Existing road damage detection models are facing challenges such as missed detection of fine cracks, poor adaptability to lighting changes, and false positives under complex backgrounds. In this study, we propose an enhanced YOLO-based framework, YOLO-ERCD, designed to improve the accuracy and robustness of sensor-acquired image data for road crack detection. The datasets used in this work were collected from vehicle-mounted and traffic surveillance camera sensors, representing typical visual sensing systems in automated road inspection. The proposed architecture integrates three key components: (1) a residual convolutional block attention module, which preserves original feature information through residual connections while strengthening spatial and channel feature representation; (2) a channel-wise adaptive gamma correction module that models the nonlinear response of the human visual system to light intensity, adaptively enhancing brightness details for improved robustness under diverse lighting conditions; (3) a visual focus noise modulation module that reduces background interference by selectively introducing noise, emphasizing damage-specific features. These three modules are specifically designed to address the limitations of YOLOv10 in feature representation, lighting adaptation, and background interference suppression, working synergistically to enhance the model’s detection accuracy and robustness, and closely aligning with the practical needs of road monitoring applications. Experimental results on both proprietary and public datasets demonstrate that YOLO-ERCD outperforms recent road damage detection models in accuracy and computational efficiency. The lightweight design also supports real-time deployment on edge sensing and control devices. These findings highlight the potential of integrating AI-based visual sensing and intelligent control, contributing to the development of robust, efficient, and perception-aware road monitoring systems.

## 1. Introduction

Road damage is a widespread problem that poses serious risks to traffic safety and driving comfort, leading to increased accident rates, accelerated vehicle wear, and higher maintenance costs [1]. Effective road damage detection and timely repair are crucial not only for ensuring road safety but also for extending the lifespan of road infrastructure [2]. However, traditional road damage detection methods heavily rely on manual inspections, which are time-consuming, labor-intensive, and prone to human error. The limitations mentioned, coupled with the lack of scalability and consistency, make manual inspections unsuitable for large-scale road monitoring tasks. Therefore, there is an urgent need for automated, efficient, and accurate road damage detection systems.

Recent advancements in computer vision and deep learning have propelled the development of image-based automated detection methods, yielding substantial improvements in both accuracy and efficiency [3,4,5]. For example, deep learning-based approaches have been applied to a wide range of transportation-related tasks, such as flight arrival time prediction [6] and speech recognition in air traffic control communications [7], further demonstrating the potential of artificial intelligence in intelligent transportation systems. Among deep learning-based object detection methods, the YOLO (You Only Look Once) series [8,9,10,11,12,13,14] stands out for its remarkable performance, especially in real-time applications. For instance, in the field of road damage detection, Haider [15] leveraged YOLO-based models to design a convolutional neural network-powered smart transportation system, enabling the accurate detection of road cracks and mitigating potential navigation hazards. Wang et al. [16] introduced YOLO-RD, which integrates several innovative modules to enhance the detection of small and irregular road damage, achieving improved performance on the Japanese road surface subset of the RDD2022 dataset [17]. Obviously, such studies highlight the effectiveness of the YOLO framework in road damage detection tasks.

Nevertheless, YOLO-based improvements such as YOLO-RD and RDD-YOLO [18] still have clear limitations in real-world road environments. Although these methods have achieved better accuracy on benchmark datasets, they are typically trained and evaluated under fixed conditions and lack explicit mechanisms to handle sudden lighting changes, complex background noise, or the preservation of elongated and fine-grained crack features. As a result, their robustness and generalization remain insufficient for practical deployment, especially in challenging scenarios where diverse lighting, weather variations, and cluttered backgrounds (e.g., buildings, pedestrians, vehicles) frequently degrade detection performance [19]. These limitations highlight the need for further optimization to enhance the adaptability and reliability of YOLO models in real- world applications.

To address the aforementioned challenges in road damage detection, this study proposes a novel architecture built upon the state-of-the-art YOLOv10 [20] framework, specifically designed to enhance detection accuracy and overall robustness in complex real-world environments. In particular, three innovative modules are introduced to strengthen feature representation, improve environmental adaptability, and effectively suppress background interference. The integration of these modules enables the proposed model to achieve significant performance gains in terms of detection accuracy and stability under challenging conditions. The main contributions of this paper can be summarized as the introduction of the following three modules:

- **Residual Convolutional Block Attention Module (R-CBAM)**: Convolutional Block Attention Module (CBAM) [21] is a popular attention mechanism that enhances feature representation by sequentially applying channel and spatial attention. However, simply stacking multiple CBAM modules does not effectively improve performance, as it can lead to feature redundancy and distortion. To address this limitation, we propose a novel design inspired by residual connections, where the original features are concatenated with the attention-refined features along the channel dimension. This approach increases the feature capacity, allowing the model to preserve original information while integrating attention-enhanced features more effectively. As a result, R-CBAM effectively mitigates feature distortion, enhances detection accuracy, and preserves a lightweight design suitable for real-time applications.
- **Channel-wise Adaptive Gamma Correction Module (CAGC)**: Inspired by the nonlinear nature of human visual perception of light intensity, we introduced gamma correction to enhance fine-grained brightness details, improving the model’s ability to detect subtle features. Building on gamma correction, we further developed an adaptive mechanism that applies channel-wise gamma adjustments with stochastic variations during training. This improvement not only simulates diverse lighting scenarios but also enhances the model’s robustness to environmental variations and challenging lighting conditions.
- **Visual Focus Noise Modulation Module (VFNM)**: Inspired by the human eye’s ability to focus on important regions, VFNM adaptively modulates Gaussian noise based on the spatial density of target objects. By reducing noise in target-dense regions and increasing it in sparse areas, this module encourages the model to focus on damage-specific features, thereby improving detection accuracy and robustness in cluttered environments.

To conlude, the design of these modules is inspired by the information processing mechanisms of the human visual system [22,23], highlighting the synergy among the three key cognitive processes of “enhancement, adaptation, and suppression.” Specifically, the R-CBAM module emulates the human eye’s ability to focus on salient spatial and channel features, thus improving the representation of subtle damage patterns; the CAGC module draws upon the eye’s adaptive response to illumination intensity, enhancing the model’s robustness to diverse lighting conditions; and the VFNM module mimics the human visual system’s dynamic allocation of attention between targets and background, using density-aware noise modulation to effectively suppress background distractions. The combined effect of these modules forms a bio-inspired visual pathway characterized by “feature enhancement–adaptive perception–robust focus”, which significantly improves detection accuracy and generalization in complex road scenarios.

## 2. Related Work

### 2.1. Traditional Methods

In the early stages of road damage detection research, traditional image processing techniques were predominantly employed. These methods typically relied on edge detection, morphological processing, threshold segmentation, and texture analysis to identify cracks and potholes on road surfaces. For instance, Canny edge detection and Sobel operators were commonly utilized to extract edge features from pavement images, while morphological operations were applied to suppress noise and reconnect fragmented edges [24,25]. However, these traditional approaches performed poorly in complex road environments, being easily affected by lighting variations, shadows, and road debris. Furthermore, traditional methods exhibited limited generalization capability when handling different types of road damage, making them incompetent for practical applications.

### 2.2. Deep Learning Methods Using CNN

Over the past decade, deep learning models have revolutionized image processing and computer vision, becoming the primary approach to the detection of road damage. These include Faster R-CNN [26] and Cascade R-CNN [27,28], known for their superior accuracy in object localization and identification. In particular, Faster R-CNN is commonly used in road damage detection [29,30], initially generating candidate boxes to focus on target objects, ensuring high accuracy and recall. On the basis of Faster R-CNN, Gou et al. [31] enhanced this model to detect cracks in complex asphalt images, while Liu et al. [32] introduced Segmentation R-CNN for comprehensive defect information, and Shen et al. [33] designed a Cascade R-CNN-based method for high precision. But all these methods suffer from high computational complexity because of their two-stage architecture, which poses challenges for real-time detection.

Single-stage algorithms, such as SSD [34] and RetinaNet [35], are renowned for their fast inference speed. They achieve detection results in a single forward pass by directly generating object-class probabilities and location coordinates. Among this category, the YOLO models are particularly popular because they approach object detection as a regression problem, and rapidly predict bounding boxes and classes using end-to-end detection. Known for their high speed and accuracy, the YOLO model family excels in various tasks. For example, Shao et al. [36] applied YOLOv3 with PTZ cameras for crack dimension measurement, Yu et al. [37] enhanced YOLOv5 with a Bottleneck Transformer for improved global information acquisition, and Roy [38] proposed the DenseSPH-YOLOv5 model, which integrates multiple feature extraction modules and improved prediction heads to address the limitations of feature extraction and multi-scale object detection accuracy in complex noisy environments. For resource-constrained scenery, which needs real-time computation, Zeng et al. [39] proposed a lightweight YOLOv8-based algorithm to optimize feature extraction while reducing computational load, and Li et al. [18] introduced RDD-YOLO, which integrates SimAM and GhostConv to increase the accuracy and efficiency for road damage detection, achieving significant performance gains over the baseline model.

### 2.3. Deep Learning Methods Using Transformer

Detection Transformers (DETRs) represent a cutting-edge, end-to-end object detection model utilizing the Transformer architecture [40]. By treating object detection as a sequence-to-sequence task, DETR eliminates the need for traditional anchor boxes and candidate regions, thereby improving both accuracy and interpretability. However, DETR faces challenges such as high computational demands, long training times, and reduced effectiveness in detecting small objects, limiting its utility in road damage detection. To solve the problem, Wan et al. [41] proposed an enhanced DETR-based model to detect bridge surface damage by refining the convolutional, attention, and feedforward layers within the encoder and decoder. Another improved version of DETR-based model is called RT-DETR [42], which is specifically designed for real-time object detection.

## 3. Method

While the aforementioned studies have substantially advanced road damage detection, further improvements in both accuracy and processing speed remain crucial for real-world applications, particularly those involving on-board camera systems. Building upon the recent advancements of YOLOv10, which has achieved remarkable breakthroughs in detection precision and computational efficiency, this study introduces an enhanced framework that integrates several customized modules. These innovative modules featuring fusion-based mechanisms and optimized architectural designs are specifically developed to improve YOLOv10’s adaptability and robustness for road damage detection under diverse and challenging environmental conditions.

### 3.1. Network Overview

Building on YOLOv10s, we propose YOLO-ERCD (You Only Look Once, Efficient Road Crack Detection), a real-time detector for on-board camera scenarios with complex backgrounds, diverse illumination, and fine-grained damage patterns. The central idea is to improve feature quality under strict latency constraints. We retain the efficient C2f-based backbone and the hybrid head of YOLOv10s, that is, many-to-one supervision during training and one-to-one inference that reduces reliance on post hoc NMS [43], so that the gains come from stronger representations rather than altered postprocessing.

**Figure 1:**
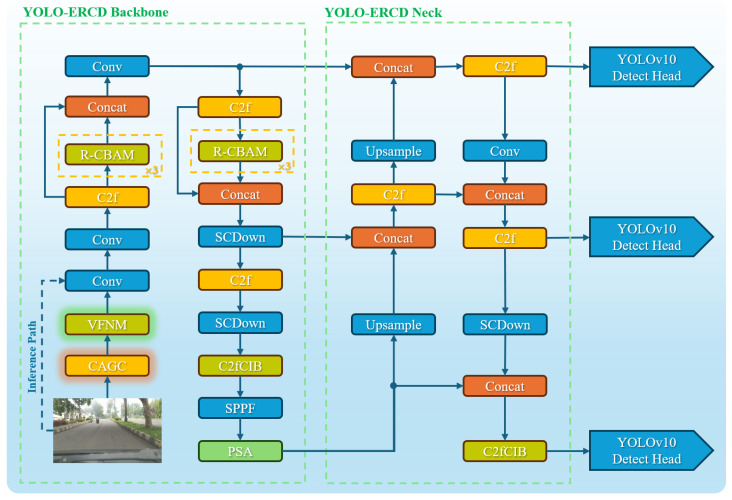
Overview of the YOLO-ERCD architecture. The model builds upon YOLOv10s, incorporating R-CBAM for enhanced spatial and channel attention, CAGC for adaptive illumination simulation, and VFNM for density-aware noise modulation. Standard components from YOLOv10s, such as C2fCIB, PSA, and SCDown, remain unchanged. The enhancements specifically target fine-grained road crack detection under challenging real-world conditions.

As shown in Figure 1, the R-CBAM module is integrated into the backbone to enhance joint channel and spatial attention during multi-scale feature extraction. This design helps preserve thin and elongated structures, such as micro-cracks, that are easily attenuated by plain feature aggregation. The residual concatenation further preserves original feature information and stabilizes the optimization process, as detailed in Section 3.2.

The other two modules are integrated into the backbone network in the same way. The CAGC module applies channel-wise stochastic gamma perturbations at the input stage during training, enhancing brightness representation and simulating realistic illumination variations, including overexposure, underexposure, and shadow conditions, without introducing inference overhead, as described in Section 3.3. The VFNM module adaptively modulates additive noise based on target density during training. This mechanism protects regions rich in damage evidence and suppresses interference from complex backgrounds and irrelevant elements, achieving improved robustness at negligible computational cost, as presented in Section 3.4.

It should be noted that the modules labeled as “C2fCIB”, “PSA”, and “SCDown” in Figure 1 are all standard components from the original YOLOv10 architecture, responsible for efficient feature extraction, spatial attention, and downsampling operations, respectively. For detailed implementations of these standard modules, please refer to the original YOLOv10 paper [20]. In this work, these components remain unchanged. The main innovations of this study are the introduction of the new R-CBAM, CAGC, and VFNM modules.

To conclude, compared with the original YOLOv10s that rely on C2f-based multi-scale representation and conventional feature fusion, YOLO-ERCD keeps the detection head unchanged and retains the overall C2f backbone topology while integrating R-CBAM to strengthen saliency-aware feature extraction; in addition, it introduces training-time input modulation for illumination and noise via CAGC and VFNM. These targeted changes address low contrast, small spatial extent, and background clutter without compromising the real-time advantage of the baseline. Ablation studies demonstrate that each changed module contributes complementary performance gains, and their integration achieves an optimal balance between detection accuracy and latency, making the framework well-suited for practical on-board applications.

### 3.2. Residual Convolutional Block Attention Module

**Figure 2:**
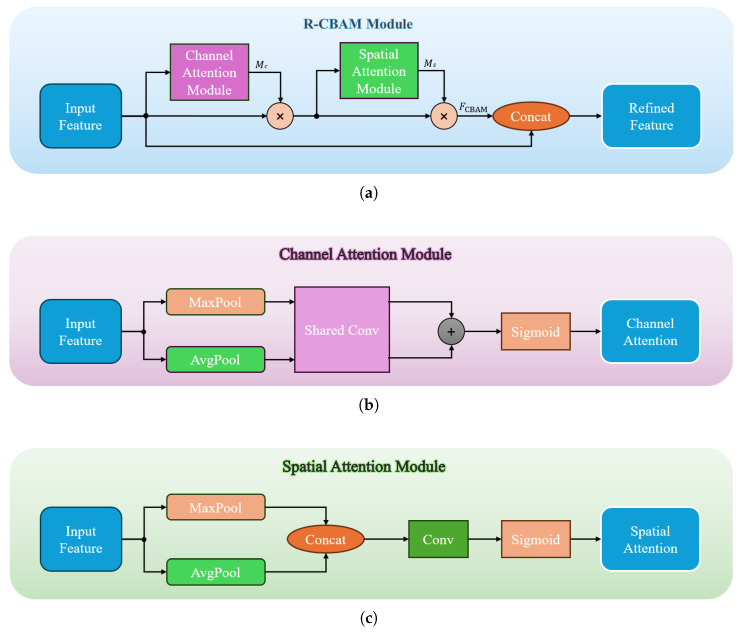
Detailed structure of the proposed Residual Convolutional Block Attention Module (R-CBAM). (**a**) Overall R-CBAM architecture, which concatenates the original feature map with the CBAM-refined features along the channel dimension to preserve essential information and enhance attention; (**b**) Channel Attention Module (CAM) for adaptive channel-wise weighting; (**c**) Spatial Attention Module (SAM) for emphasizing informative spatial locations.

As shown in Figure 2, the R-CBAM module consists of two sub-modules: the Channel Attention Module (CAM) and the Spatial Attention Module (SAM), which are connected sequentially to jointly refine the feature maps.

Specifically, the CAM module enhances the feature representation of each channel by adaptively weighting its importance. This is accomplished by applying global max pooling and global average pooling to each channel of the input feature map, followed by a shared convolutional layer to generate the attention map, as illustrated in Figure 2b. The kernel size of the shared convolution layer is 1×1, and its parameters are shared across spatial locations, modeling the relationship only along the channel dimension. This is similar to a fully connected layer that linearly integrates global channel information. The operation is defined as

(1) SharedConv(·)=Conv(ReLU(Conv(·)))

The second module, namely SAM, highlights the importance of specific spatial locations by aggregating multi-scale contextual information. It concatenates the max-pooled and average-pooled feature maps along the channel dimension, followed by a convolutional layer to generate spatial attention weights, as shown in Figure 2c. The concatenation operation merges these pooled features into a unified representation that captures both global maximum and average contextual cues, thereby enhancing spatial feature discrimination.

Then, the CBAM feature $F_{\mathrm{CBAM}}$ is obtained by sequentially applying the channel and spatial attention maps to the input feature map *F*:

(2) $$F_{\mathrm{CBAM}}=F⊙M_{c}⊙M_{s},$$

where $F\in R^{C\times H\times W}$ is the input feature map, $M_{c}\in R^{C\times 1\times 1}$ is the channel attention map, $M_{s}\in R^{1\times H\times W}$ is the spatial attention map, and ⊙ denotes element-wise multiplication with broadcasting along singleton dimensions.

While CBAM effectively enhances feature attention, we observed that excessively stacking CBAM layers can lead to performance degradation. This occurs because repeated attention operations may distort original feature representations and increase training difficulty. To mitigate this issue, we propose the R-CBAM, which introduces a residual connection by concatenating the original feature map *F* with the CBAM-enhanced features $F_{\mathrm{CBAM}}$. This integration allows the model to preserve essential information from the original feature map while simultaneously benefiting from the refined attention features. The operation is defined as follows:

(3) $$F_{R-\mathrm{CBAM}}=\mathrm{Concat}\left(F,F_{\mathrm{CBAM}}\right)$$

Here, the concatenation is performed along the channel dimension. By retaining the original features *F* alongside the attention-enhanced features $F_{\mathrm{CBAM}}$, R-CBAM mitigates feature distortion, alleviates gradient vanishing, and reduces the risk of overfitting, thereby improving model stability and overall performance.

Beyond qualitative benefits, it is also important to quantitatively assess the computational impact of these modifications. Table 1 compares the number of parameters and FLOPs between YOLOv10s and YOLO-ERCD. Each additional stacked R-CBAM layer increases the parameter count by approximately 0.016M and the FLOPs by about 0.005 GFLOPs. Since R-CBAM concatenates the original features with the attention-refined features, the channel number is doubled, which requires the subsequent convolutional layer to accommodate the increased channel dimension. For example, if the input feature map *F* to the first R-CBAM has C=128 channels, the output will have 2C=256 channels, while the spatial dimensions remain unchanged. To ensure structural compatibility, the subsequent convolutional layer’s input channels are set to 256, with other parameters remaining consistent with the original YOLOv10 backbone. Statistically, stacking three R-CBAM layers results in a total parameter increase of about 0.12M, accounting for approximately 2.1% of the total parameters of YOLOv10s. The corresponding increase in computation is about 1.4 GFLOPs, or 5.6% of the total computation, which has only a minor impact on overall inference efficiency. The concatenation operation itself does not introduce new parameters, but it effectively improves.

### 3.3. Channel-Wise Adaptive Gamma Correction Module

As established by classical psychophysical research [44], human visual perception of brightness is nonlinear and adaptive, following a power–law relationship with physical intensity. Inspired by this biological mechanism, the CAGC module is designed based on gamma correction [45], a classical nonlinear image processing technique used to adjust image brightness and contrast according to the human visual system. The transformation is defined as follows:

(4) $$I^{\prime }=A\cdot I^{\gamma },$$

where *A* is a constant, *I* and $I^{\prime }$ represent normalized input and output image, respectively, and the parameter γ controls the degree of brightness adjustment. When γ > 1, the darker regions of the image are compressed, resulting in an overall darker appearance. This adjustment enhances details in bright areas by reducing the dominance of highlights. Conversely, when γ < 1, the brighter regions are compressed, making the image globally lighter and amplifying subtle details in darker regions.

**Figure 3:**
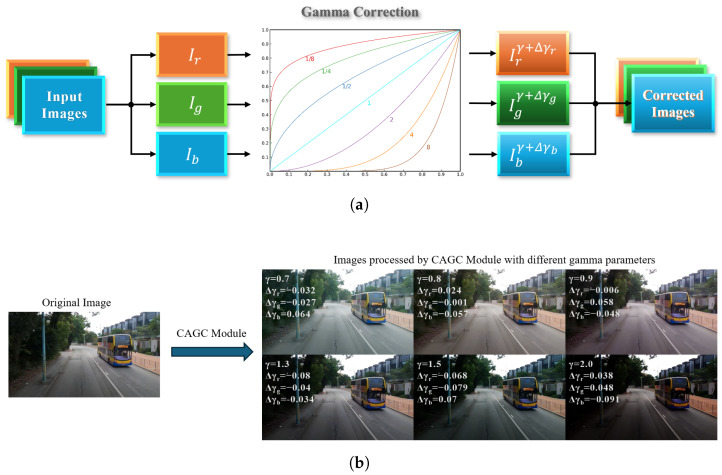
Visualization of the Channel-wise Adaptive Gamma Correction (CAGC) module. (**a**) Workflow of CAGC: input images are separated into RGB channels, and each channel undergoes gamma correction with a stochastically perturbed exponent to simulate diverse illumination conditions before being recombined as corrected images. (**b**) Example results: the left shows the original image, and the right demonstrates CAGC-processed images under different gamma settings, illustrating how channel-wise adaptive gamma perturbation enhances both bright and dark details for improved robustness to lighting variations.

Classical gamma correction applies a uniform γ value across all RGB channels to enhance overall image brightness and contrast. To address the challenges of road damage detection under diverse and complex lighting conditions, we propose the Channel-wise Adaptive Gamma Correction (CAGC) module, as illustrated in Figure 3. The CAGC module introduces stochastic variations in γ during training to simulate real-world illumination effects such as shadows, reflections, and ambient light fluctuations. Specifically, instead of applying a fixed γ value across all channels, CAGC perturbs the base value with small, random deviations for each channel as follows:

(5) $$I^{\prime }=\mathrm{Concat}\left(A\cdot I_{r}^{\gamma +\Delta \gamma_{r}},\;A\cdot I_{g}^{\gamma +\Delta \gamma_{g}},\;A\cdot I_{b}^{\gamma +\Delta \gamma_{b}}\right),$$

where $I_{c}\in {\left[0,\;1\right]}^{H\times W}$ (c∈{r,g,b}) denotes the normalized input image in each channel, *A* is a brightness scaling constant, γ is the base gamma value, and $\Delta \gamma_{c}$ represents a small, random variation unique to each channel.

Here, *A* is generally set to 1, as in conventional gamma correction, so the output brightness is determined solely by γ; if A>1, it linearly increases overall brightness, which can be useful for specific scenarios. In this study, we fix A=1 for simplicity, but retain this parameter for potential future extensions involving joint brightness and contrast enhancement.

The base γ is randomly sampled from the interval [0.45, 2.2] for each training batch, which is set to cover typical scenarios of underexposure and overexposure encountered in real-world road images. For each channel, $\Delta \gamma_{c}$ is independently sampled from [−a,a], where a=0.1γ. This proportional setting ensures that the perturbation magnitude is scaled with the base γ, preventing excessive perturbation when γ is small and insufficient enhancement when γ is large. In practice, setting a=0.1γ provides a reasonable trade-off between augmentation diversity and stability. This dynamic sampling mechanism enables the model to adaptively generalize to various lighting scenarios, thereby improving the robustness of road damage detection and enhancing the diversity of the training data distribution.

The CAGC module functions as both a feature enhancement mechanism and a data augmentation strategy. By emphasizing brightness-related details and simulating environmental variability, it enhances the model’s robustness under diverse lighting conditions, weather scenarios, and road surface textures. As illustrated in Figure 3b, the CAGC-processed images exhibit improved visibility of road damage while maintaining realistic ambient lighting effects. Notably, the CAGC module is applied exclusively during training and does not influence inference speed, thereby preserving the computational efficiency of the YOLOv10 framework.

### 3.4. Visual Focus Noise Modulation Module

**Figure 4:**
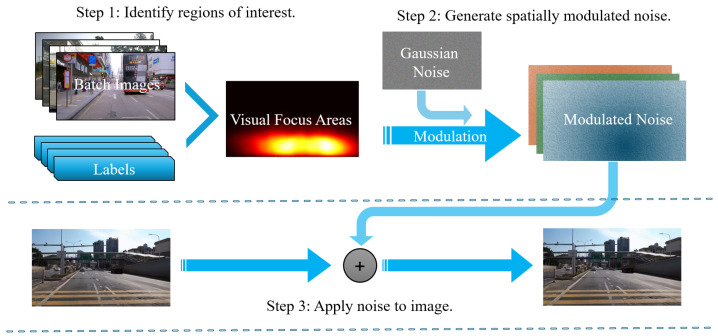
Workflow of the Visual Focus Noise Modulation (VFNM) module. Step 1: Regions of interest are identified from batch images and labels based on target density, generating a visual focus map. Step 2: Gaussian noise is spatially modulated according to the focus map, producing noise with reduced intensity in target-dense areas and stronger intensity in background regions. Step 3: The modulated noise is selectively applied to the input image, enhancing model robustness by protecting critical features while regularizing background areas.

Deep learning models often struggle to maintain robustness in complex environments, particularly in the presence of cluttered backgrounds. Conventional noise injection techniques, although effective for regularization, fail to consider the spatial distribution of target objects and may introduce unnecessary interference in critical regions. Inspired by the human visual mechanism where attention is concentrated on salient targets while background details are de-emphasized, we propose the VFNM module to overcome these limitations. VFNM enhances model robustness by adaptively modulating noise across image regions according to target density, thereby preserving essential features in target areas while suppressing irrelevant background information. As illustrated in Figure 4, the overall structure of VFNM comprises three main components: (1) identifying areas of interest based on target density, (2) generating modulated noise, and (3) applying the noise to input images. The detailed processes of these steps are described below.

To operationalize the abovementioned concept, the VFNM module utilizes statistical information derived from the training data to identify areas of interest based on target density. By concentrating on regions with a high concentration of target objects, VFNM ensures that noise modulation is spatially aligned with the distribution of critical features. Specifically, consider a batch containing *n* bounding boxes, where each box is described as $B_{i}=\left(x_{i},y_{i},w_{i},h_{i}\right)$, with $x_{i},y_{i}$ representing the center coordinates, and $w_{i},h_{i}$ denoting the width and height of the bounding box. For target focus area, a function T(x,y) is defined to represent the aggregated density of target objects at each spatial location (x,y) within the image:

(6) $$T\left(x,y\right)=\sum_{i=1}^{n}I\left(\left(x,y\right)\in B_{i}\right),$$

where I(·) represents the indicator function, the value of which equals one in case the point (x,y) lies within the bounding box $B_{i}$; otherwise, the value will be set to zero. In this study, the statistical range of the target density function T(x,y) covers all target bounding boxes within one training batch, which allows the use of intra-batch target distribution information (for example, the spatial distribution patterns of road cracks captured by an on-board camera) to enhance the model’s adaptability to real-world crack distributions. For special datasets, the number of images included in the statistics can also be flexibly adjusted.

To ensure that the resulting distribution is comparable across images, T(x,y) is normalized to $\hat{T}\left(x,y\right)$, which highlights the relative density of target objects and serves as a key factor in modulating noise intensity. Specifically, the modulated noise $N_{\mathrm{mod}}\left(x,y\right)$ is generated as follows:

(7) $$N_{\mathrm{mod}}\left(x,y\right)=\left(1-\alpha \cdot \hat{T}\left(x,y\right)\right)\cdot N_{\mathrm{gauss}}\left(x,y\right),$$

where $N_{\mathrm{gauss}}\left(x,y\right)∼N\left(\mu ,\sigma^{2}\right)$ denotes Gaussian noise with mean μ and standard deviation σ, and α is a scaling parameter that controls the influence of target density, with a value range from 0 to 1. When α=0, VFNM degenerates into standard Gaussian noise injection, applying noise uniformly to the whole image; when α=1, no noise is added to the target regions, and only background areas are injected with noise. In this study, we set α=0.8 to balance the protection of target region features and the regularization of background regions.

The modulation in Equation (7) attenuates noise intensity in target-dense regions (where $\hat{T}\left(x,y\right)$ is high) and amplifies it in sparse regions (where $\hat{T}\left(x,y\right)$ is low), thereby aligning with the module’s objective of emphasizing critical areas.

The final modulated image $I_{\mathrm{mod}}\left(x,y,c\right)$ is computed as

(8) $$I_{\mathrm{mod}}\left(x,y,c\right)=\left\{\begin{array}{cc} I\left(x,y,c\right)+N_{\mathrm{mod}}\left(x,y\right), & \mathrm{if}\;c\in C,\\ I(x,y,c), & \mathrm{otherwise}, \end{array}\right.$$

where $I\in R^{H\times W\times C}$ is the input image, $T,\hat{T},N_{\mathrm{gauss}},N_{\mathrm{mod}}\in R^{H\times W}$ are spatial maps, and C⊆{1,2,…,C} is the set of selected channels.

To sum up, the process ensures that the modulated noise is applied selectively, with varying intensities tailored to both target-dense and background regions. In summary, VFNM adaptively modulates noise intensity across input images, enhancing model robustness in noisy or complex environments by preserving critical features within target-dense regions and suppressing irrelevant background interference.

## 4. Results and Discussion

### 4.1. Evaluation Metric

To evaluate the model’s performance, we employed mean average precision (mAP), a widely used metric in object detection tasks. Specifically, we focused on mAP@0.5, where “@0.5” denotes an Intersection over Union (IoU) threshold of 0.5. The IoU measures the overlap between the predicted bounding box (*P*) and the ground truth bounding box (*G*), and is defined as follows:

(9) $$IoU=\frac{Area_{\mathrm{Overlap}}}{Area_{\mathrm{Union}}}=\frac{\left|P\cap G\right|}{\left|P|+|G|-|P\cap G\right|},$$

where |P∩G| represents the area of overlap between the two bounding boxes, while |P|+|G|−|P∩G| is the area of their union.

To calculate mAP, we need Average Precision (AP) first, which represents the area under the precision–recall curve for a given class. Specifically, Precision is defined as the ratio of true positive detections to the total number of positive detections, whereas recall (r) is the ratio of true positive detections to the total number of actual positives. The AP is calculated as follows:

(10) $$\mathrm{AP}=\int_{0}^{1}\mathrm{Precision}\left(r\right)\;dr$$

In road damage detection scenarios, the IoU threshold is typically set to 0.5, reflecting the relatively high tolerance for boundary variations in crack annotations. Accordingly, mAP@0.5 evaluates the model’s detection accuracy at this threshold, where higher mAP@0.5 values indicate superior overall performance.

### 4.2. Dataset

**Figure 5:**
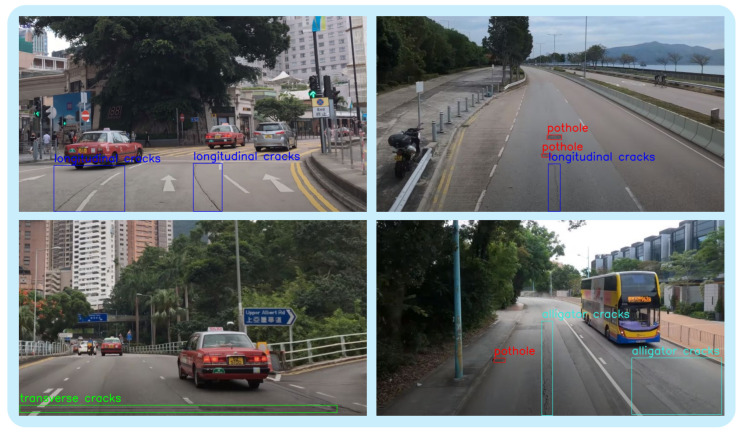
Sample images in the HKPC dataset.

This study employs a dataset provided primarily by the Hong Kong Productivity Council (HKPC). The images were captured across various locations in Hong Kong using both personnel-operated and on-board cameras and were standardized to a resolution of 1920 × 1080. The dataset was annotated and classified into four categories of road damage based on their distinct characteristics: pothole, alligator cracks, transverse cracks, and longitudinal cracks (as illustrated in Figure 5). In total, the dataset comprises 9630 images, which were divided into training, validation, and test sets in an 8:1:1 ratio. The category distribution of road damage types within the HKPC dataset is summarized in Table 2. It should be noted that, since a single image may contain multiple damage targets, the number of annotations typically exceeds the number of images. The “number of samples” in Table 2 refers to the total number of annotated bounding boxes for each damage category across all images. Overall, the dataset encompasses a wide variety of road damage patterns, providing comprehensive coverage to support effective model training and evaluation.

To further validate the robustness and generalizability of our model, we also employed the RDD2022 dataset [17] from the Crowdsensing-based Road Damage Detection Challenge (CRDDC’2022) [46]. This dataset comprises 38,385 images collected from diverse geographical regions, including Japan, India, the Czech Republic, Norway, the United States, and China, making it well-suited for evaluating the generalization performance of road damage detection models.

**Figure 6:**
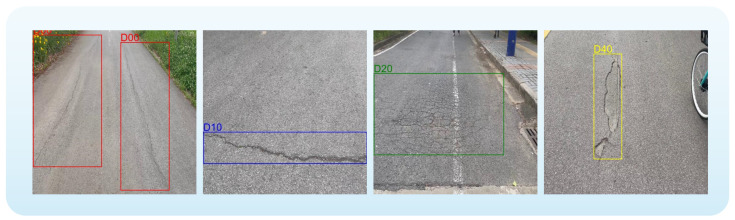
Sample images in the RDD2022 dataset, from left to right: longitudinal cracks (D00), transverse cracks (D10), alligator cracks (D20), and potholes (D40).

To ensure data consistency and experimental reproducibility, we performed cleaning and preprocessing on the original data: first, extremely rare damage categories were removed, retaining only the four major types of damage: longitudinal cracks (D00), transverse cracks (D10), alligator cracks (D20), and potholes (D40), as illustrated in Figure 6. Next, since the original image sizes varied, all images were normalized to a unified size of 640 × 640 using scaling and cropping operations; finally, images with invalid or missing annotations were further checked and removed. All processing steps were strictly implemented to ensure fairness and validity in subsequent experiments. The distribution of images for each damage category is summarized in Table 3.

In this study, the RDD2022 dataset was split into training, validation, and test sets in a 70%/15%/15% ratio. To enhance the generalization ability of the model, a globally randomized splitting strategy was adopted, ensuring that each subset contains samples from different geographical regions. The reason for choosing random splitting instead of grouping by geographic distribution is that the road environments in different regions vary significantly in terms of material, damage types, and background conditions. Allowing the model to access diverse road styles during training facilitates the learning of more generalizable feature representations, improves adaptability to complex real-world scenarios, and helps to prevent overfitting to specific regions.

### 4.3. Experimental Setup

The experiments were conducted on a Linux operating system (Ubuntu 22.04.3 LTS) using Python 3.12.0 and the PyTorch 2.4.0 deep learning framework, running on an NVIDIA A100 Tensor Core GPU (Nvidia, Santa Clara, CA, USA) with 48 GB of memory. The Stochastic Gradient Descent (SGD) optimizer was employed with an initial learning rate of 0.01, a batch size of 32, a momentum of 0.9 to accelerate convergence, and a weight decay of 0.0005 for regularization.

Due to the class imbalance present in the datasets, specifically the fact that both the HKPC and RDD2022 datasets contain a significantly larger number of pothole samples compared to other categories, a class-adaptive data augmentation strategy was adopted during the data augmentation stage. For categories with fewer samples, a higher proportion of data augmentation was applied so that the number of samples for each class became more balanced after augmentation. This approach is intended to enhance the model’s detection performance for minority classes. The additional data augmentation operations used in this study included horizontal flipping and cropping. Since the orientation of the on-board camera is fixed, vertical flipping was not used in order to retain the spatial distribution characteristics of road damage.

### 4.4. Performance Comparison

To assess the effectiveness of the proposed model, we conducted comparative experiments against several state-of-the-art object detection networks for road damage detection using the HKPC and RDD2022 datasets. The baseline models included Faster R-CNN [26], RT-DETR [42], YOLOv5 [47], YOLOv8 [43], RDD-YOLO [18], and YOLOv10 [20], as well as the most recent YOLOv11 [48] and YOLOv12 [49] models that were released after the completion of our main experiments. As summarized in Table 4, the proposed model (YOLO-ERCD) achieved the highest performance among all evaluated methods.

Specifically, on the HKPC dataset, YOLO-ERCD achieved a mAP@0.5 score of 0.677, which is 2.1 percentage points higher than the baseline YOLOv10s and also slightly better than the subsequently released YOLOv11 and YOLOv12 models. However, considering that the new YOLOv11 baseline demonstrates even stronger performance for road damage detection, we believe that future work based on YOLOv11 can achieve further improvements. On the RDD2022 dataset, YOLO-ERCD also slightly outperformed other baseline methods and reached performance comparable to the latest YOLOv11 and YOLOv12 models. Despite these improvements in accuracy, the computational cost of the proposed model remains efficient, with a GFLOPs value of 26.2, which is only 1.4 higher than YOLOv10s. The number of parameters is 8.2M, just 0.1M more than YOLOv10s, ensuring that inference speed is not affected.

To comprehensively evaluate the stability of the model improvements, we conducted independent repeated training of our method with five different random seeds under the same configuration. On the HKPC dataset, the mean mAP@0.5 was 0.6768 with a standard deviation of 0.0009, and the lowest result was 0.675. The corresponding coefficient of variation (CV) was 0.13%. On the RDD2022 dataset, the mean mAP@0.5 was 0.6406 with a standard deviation of 0.0012, and the lowest result was 0.638, with a CV of 0.19%. These low coefficients of variation indicate that the model’s performance fluctuates minimally under different random initializations. Overall, YOLO-ERCD achieves high detection accuracy and stability while maintaining computational efficiency, making it suitable for deployment in real-world road scenarios.

Additionally, as shown in Table 4, the YOLOv8n model achieves the lowest GFLOPs, resulting in the fastest detection speed and relatively good accuracy. This makes it suitable for deployment on edge devices with extremely limited computational resources, such as legacy-embedded hardware or micro-drones. However, lightweight models based on YOLOv10s, including YOLO-ERCD, deliver significantly better detection accuracy and stronger robustness with only a slight increase in computation, which makes them more suitable for mainstream edge computing platforms, such as in-vehicle terminals or industrial cameras, where real-time processing and high accuracy are required. Overall, the “n” type models are more suitable for scenarios where resource constraints are extremely strict and moderate accuracy is acceptable, while “s” type models and our proposed model achieve a more optimal balance of accuracy and efficiency for mainstream deployment environments. Testing results show that, on a laptop equipped with an RTX4060 GPU (NVIDIA Corporation, Santa Clara, CA, USA), YOLO-ERCD achieves an average inference time of 6.6 ms per image with an input size of 640 × 640. The preprocessing and postprocessing times are 1.4 ms and 1.0 ms, respectively, resulting in an overall average processing latency of approximately 9.0 ms per image, corresponding to an inference speed of about 111 FPS. This demonstrates the real-time capability and suitability of the model for lightweight deployment. With the continuous improvement in hardware performance, selecting models with higher accuracy has become a trend. However, in ultra-low-power scenarios, users can still select ultra-lightweight models according to practical needs.

### 4.5. Ablation Study

To evaluate the contribution of each component to the overall model performance, ablation experiments were conducted in the HKPC dataset, using YOLOv10s as the baseline model. The results, summarized in Table 5, demonstrate the effects of the R-CBAM, CAGC, and VFNM modules, both individually and in combination. Each module independently improved detection precision, with VFNM and R-CBAM yielding the highest individual gains of +0.009 each. Notably, the integration of all three modules achieved the best performance, increasing mAP@0.5 from 0.656 to 0.677.

However, although both the R-CBAM and CAGC modules can independently improve model performance, the combined improvement when these two modules are used together is limited. There are several reasons for this observation. First, both R-CBAM and CAGC are designed to enhance features and improve model robustness, and both contribute to better handling of illumination variations and fine-grained feature modeling. This leads to some degree of overlap or saturation in their effects when used together. Second, when input enhancement (CAGC) and intermediate feature enhancement (R-CBAM) are jointly applied, the resulting feature distribution may change substantially, making it challenging for the model to fully utilize all information and potentially causing a certain degree of information redundancy. In summary, although the combination of these two modules still outperforms using a single module, the overall improvement is relatively limited. This finding is consistent with the typical phenomenon of diminishing returns when integrating multiple modules into a single architecture.

#### 4.5.1. Impact of the R-CBAM Module

The evaluation of the CBAM and R-CBAM modules was conducted in two stages. First, we analyzed the effect of integrating CBAM into different positions within the YOLOv10s architecture, specifically the backbone and neck. As presented in Table 6, incorporating CBAM into the backbone yielded a greater improvement in mAP@0.5 (0.661) compared to its placement in the neck (0.659). Based on these findings, all subsequent experiments were performed with the modules inserted into the backbone.

In the second stage, we compared the performance of CBAM and R-CBAM integrated into the backbone by varying the number of stacked layers, as summarized in Table 7. It was observed that when stacking a large number of standard CBAM modules, the model exhibited a notable decrease in mAP, indicating a risk of excessive feature representation and overfitting. In contrast, R-CBAM employs a channel-wise concatenation strategy that directly fuses the original features with the attention-enhanced features along the channel dimension. This approach enhances salient feature representation while effectively retaining the original information, which significantly alleviates the performance drop caused by over-stacking. As a result, R-CBAM demonstrates better stability and resistance to overfitting. These characteristics are particularly important for very deep networks or scenarios involving multi-scale feature aggregation, and they contribute to improved generalization and training stability.

Notably, R-CBAM achieved the highest mAP@0.5 of 0.665 with three stacked layers, outperforming CBAM under the same configuration. These results demonstrate the superior effectiveness of R-CBAM in improving model accuracy while preserving network efficiency.

#### 4.5.2. Impact of the CAGC Module

Three experiments were conducted to evaluate the impact of different gamma correction strategies on model performance, as summarized in Table 8. Without gamma correction, the baseline model achieved an mAP@0.5 of 0.656. Introducing random gamma correction slightly improved the mAP@0.5 to 0.658, suggesting modest gains from stochastic brightness adjustments. In contrast, the proposed CAGC module further increased the mAP@0.5 to 0.662, demonstrating a clear advantage over the other strategies.

#### 4.5.3. Impact of the VFNM Module

Experiments were conducted to compare the performance of the VFNM module with conventional Gaussian noise augmentation under different noise levels, as summarized in Table 9. The noise levels were controlled by adjusting the standard deviation (σ) of the Gaussian noise. At lower noise levels, both methods enhanced model performance, with VFNM slightly outperforming Gaussian noise augmentation. As the noise level increased, the performance of Gaussian noise augmentation degraded substantially, failing to sustain effective training under high-noise conditions. In contrast, VFNM achieved its peak mAP@0.5 of 0.665 at a moderate noise level and maintained stable accuracy even under higher noise intensities.

#### 4.5.4. Module Trade-Off Analysis

To further highlight the distinctions and trade-offs between the innovative modules proposed in this work and existing approaches, this section systematically compares the design concepts, performance improvements, and computational efficiency of R-CBAM, CAGC, and VFNM. First, R-CBAM introduces a residual connection based on the traditional CBAM, concatenating the original features and attention-enhanced features along the channel dimension. This design alleviates problems such as feature redundancy and distortion, thereby enhancing feature representation capability. Compared to models without CBAM and those with the standard CBAM, R-CBAM improves mAP@0.5 on the HKPC dataset by 0.9 and 0.4 percentage points, respectively, with only a slight increase of 0.1M in parameters and 1.4 GFLOPs in computational cost. The inference speed remains sufficient for real-time applications.

Second, the CAGC module adopts a channel-adaptive gamma correction mechanism that simulates the nonlinear perception of light intensity by the human eye. During training, the gamma values of each channel are randomly perturbed, which more realistically reflects diverse illumination conditions. Compared to traditional linear illumination enhancement methods, CAGC achieves an improvement of approximately 0.6 percentage points in mAP@0.5, with almost no additional computational burden, ensuring both efficiency and illumination robustness.

Finally, VFNM adaptively adjusts the intensity of Gaussian noise based on the spatial distribution of objects within the image, allowing the model to focus on critical feature regions and avoiding the training interference commonly caused by global noise augmentation. Experiments show that, under moderate noise intensity, VFNM achieves at least a 0.6 percentage point improvement in mAP@0.5 over standard Gaussian noise augmentation, and maintains stable training performance even at higher noise levels. This module introduces only a minimal computational increase during data preprocessing, with almost no additional overhead during inference.

In summary, all three innovative modules significantly improve detection accuracy and robustness while having negligible impact on computational efficiency. This achieves a favorable balance between detection performance and inference efficiency, making the proposed model highly suitable for deployment in real-world road scenarios.

### 4.6. Category-Wise Performance Analysis

**Figure 7:**
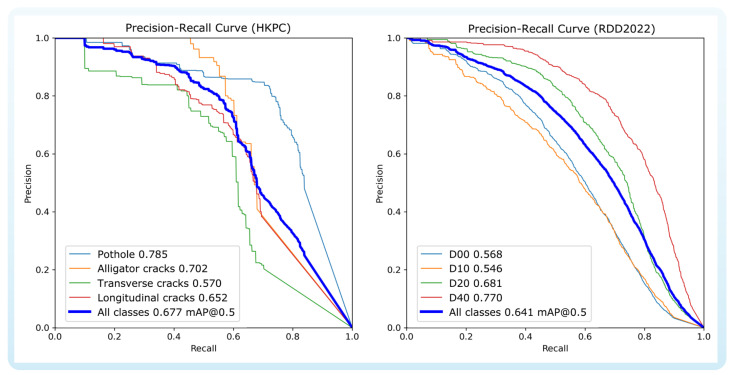
Precision–Recall curves by category (HKPC and RDD2022 dataset).

We further evaluated the model’s detection performance across different damage categories on both the HKPC and RDD2022 test sets. The detailed results are presented in Table 10 and Table 11, respectively. The Precision–Recall (P-R) curves for each category are shown in Figure 7. By comparing the results across the HKPC and RDD2022 datasets, it is evident that the model exhibits consistent trends in all categories, which suggests a certain degree of cross-dataset stability. Taking the HKPC dataset as an example, the model achieved the best performance on the pothole category, with an mAP@0.5 of 0.785, demonstrating strong detection capability for this type of damage. In contrast, the transverse cracks category showed the lowest detection accuracy, with an mAP@0.5 of 0.570.

To address the relatively low detection performance for transverse cracks, two potential improvements are proposed for future work. First, deformable convolution can be introduced, which learns spatial sampling locations for convolutional kernels, enabling the network to adaptively focus on the actual shapes and structures of the targets. This enhancement is expected to improve feature extraction for elongated or irregularly shaped targets such as transverse cracks. Second, an adaptive anchor mechanism could be designed, for instance, by dynamically adjusting the aspect ratio distribution of anchor boxes or employing anchor-free methods so that the detection head directly regresses the object contours without relying on fixed anchors. These approaches can better cover and adapt to target regions with large variations in aspect ratio, thereby improving detection performance in special categories such as transverse cracks. These structural optimizations are expected to enhance the model’s generalization ability to categories with significant aspect ratio changes and further improve the overall robustness of detection.

### 4.7. Qualitative Error Analysis

**Figure 8:**
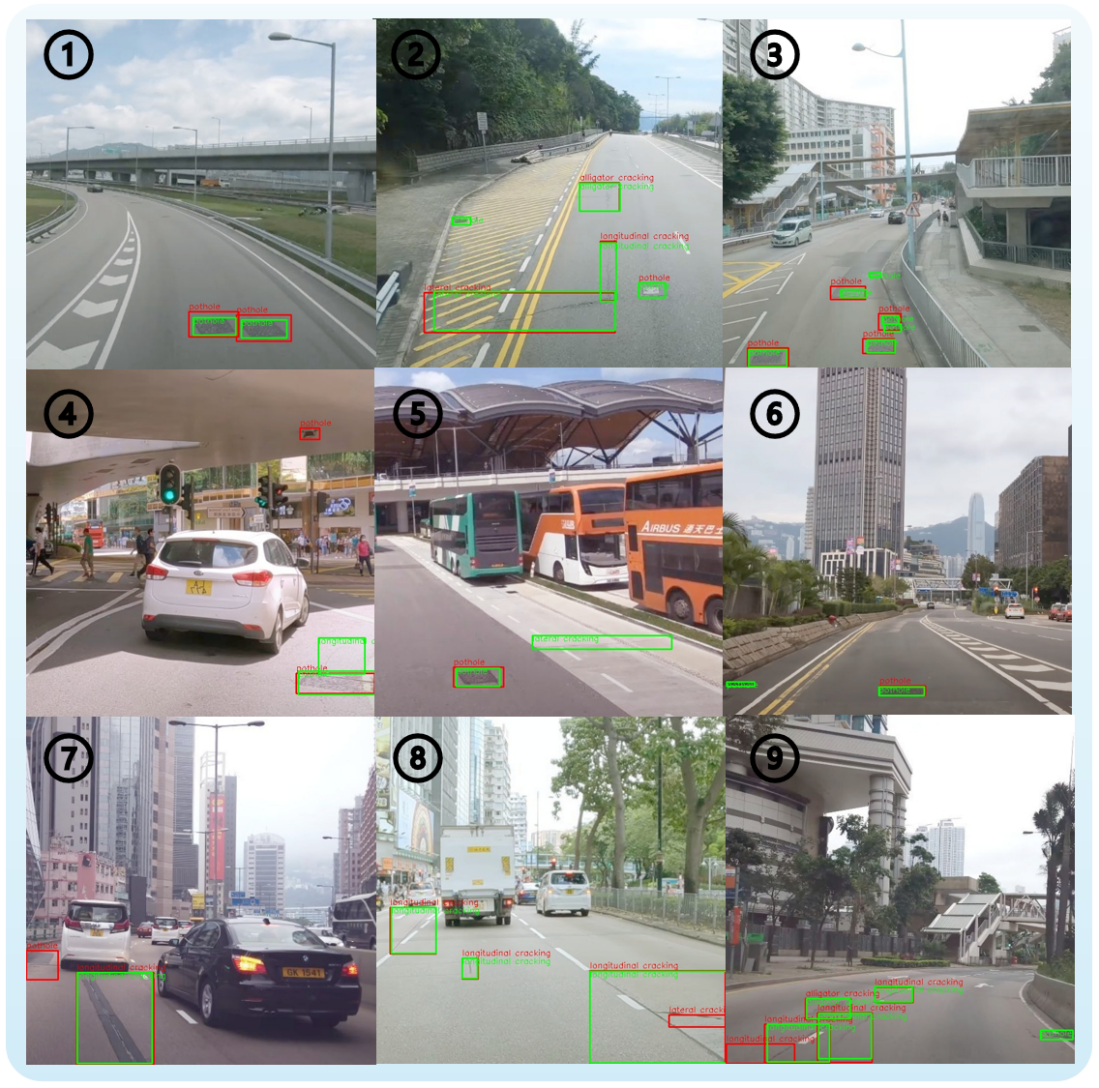
Qualitative analysis of typical false positive and false negative cases on the HKPC test set. Green boxes indicate ground truth, and red boxes represent model predictions. Each numbered example highlights a specific failure pattern related to blurred boundaries, complex morphology, low contrast, or texture interference.

To gain a more comprehensive understanding of the model’s limitations, a qualitative analysis of representative false positive and false negative cases was conducted using the HKPC dataset. As illustrated in Figure 8, green bounding boxes indicate ground truth annotations, while red bounding boxes denote model predictions. By examining the typical numbered cases, the main error patterns of the model can be summarized as those arising in scenarios with blurred boundaries, complex morphologies, and low contrast.

For example, cases 1, 2, and 3 in Figure 8 reflect challenges related to object localization. In case 1, the vague transition between a pothole and its surrounding environment leads to a noticeable offset in the predicted bounding box. In case 2, the transverse crack displays significant variation in length and a narrow width, making it difficult for the model to accurately determine the start and end positions, especially when the crack is very thin or the image is unclear. Case 3 demonstrates that two adjacent potholes were mistakenly detected as a single large pothole, indicating that the model’s ability to distinguish between irregular shapes or densely clustered targets is limited.

Regarding missed detections, cases 4, 5, and 6 are representative. In case 4, a longitudinal crack is not successfully detected under strong lighting or overexposure, as the model fails to capture its texture features. Case 5 shows that transverse cracks, due to their slender structure and low contrast, are the most likely to be overlooked. In case 6, a pothole photographed from a specific angle appears flattened, resulting in insufficient feature expression and subsequent detection failure.

In terms of false positives, cases 7, 8, and 9 illustrate the model’s shortcomings in handling pavement texture interference and complex crack structures. In case 7, a dark area on the pavement is incorrectly identified as a pothole, suggesting that the model’s ability to distinguish between texture and illumination variations needs further improvement. Case 8 demonstrates that branched cracks are sometimes detected as multiple independent targets, which may be due to the model’s limitations in recognizing complex structures or inconsistencies in annotation standards. In case 9, part of a crack is detected as a separate target, highlighting the model’s inadequate understanding of continuous object boundaries.

In summary, the analysis of these typical cases reveals that the model predominantly makes systematic errors in scenarios with ambiguous object boundaries, complex structures, small target sizes, or low contrast. Future improvements may include multi-scale feature fusion, incorporation of attention mechanisms, and more standardized data annotation practices to further enhance detection robustness and generalization capability in complex scenes.

### 4.8. Module Interpretability and Attention Distribution Analysis

**Figure 9:**
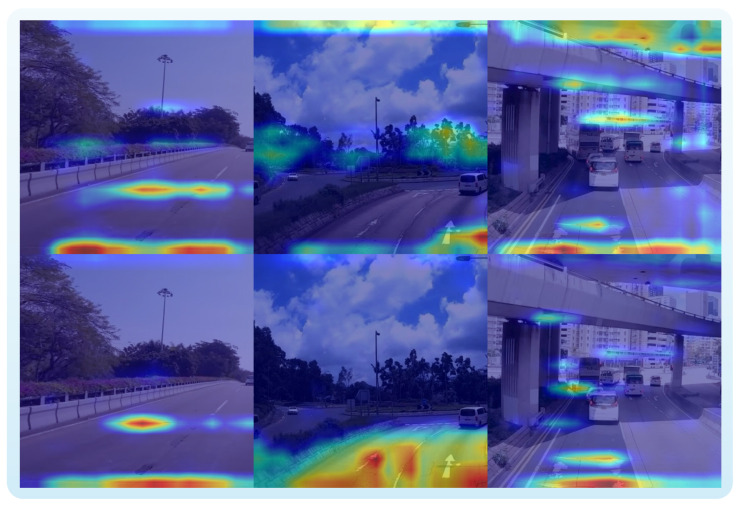
Grad-CAM visualization of attention heatmaps for the same input image. Warmer colors (e.g., red) indicate regions with higher attention, while cooler colors (e.g., blue) indicate lower attention. The first row corresponds to the baseline YOLOv10s model, while the second row displays the improved YOLO-ERCD model with CAGC, R-CBAM, and VFNM modules. The YOLO-ERCD model demonstrates more focused and accurate attention on pavement damage regions, illustrating its enhanced interpretability and detection robustness.

To further verify the contribution of the proposed CAGC, R-CBAM, and VFNM modules to model interpretability, we performed a comparative analysis of key region attention using the Grad-CAM [50] method before and after integrating these modules. Figure 9 shows activation heatmaps for the same input image, where the first row corresponds to the baseline YOLOv10s model and the second row presents results from the improved YOLO-ERCD model.

The experimental results indicate that, prior to improvement, the activation regions of the baseline model are relatively scattered, with some attention erroneously assigned to non-target areas such as flower beds and trees, reflecting a certain degree of incorrect association. In contrast, the YOLO-ERCD model demonstrates significantly enhanced adaptability to illumination changes and complex features, resulting in increased attention to pavement crack regions. In particular, after introducing the VFNM module, the model is able to adaptively adjust its attention distribution in scenarios with dense targets or complex backgrounds, focusing more distinctly on damage-related areas and effectively suppressing responses to irrelevant regions. Overall, the improved model is able to more precisely focus on road surfaces and crack distribution areas, with high-response regions in the heatmaps closely matching the actual damage locations. These findings not only enhance the interpretability of the model, but also further confirm the practical effectiveness of modules such as VFNM in improving detection accuracy and robustness.

## 5. Conclusions

This study presents an enhanced object detection model based on the YOLOv10 framework, integrating principles of human visual perception to improve the detection of road damage, especially crack. The key innovations residual convolutional block attention module, channel-wise adaptive gamma correction module, and visual focus noise modulation module jointly enhance the model’s ability to capture complex features and adapt to diverse road conditions. Ablation experiments conducted on the HKPC dataset confirmed the effectiveness of these modules, while comparative evaluations on the HKPC and RDD2022 datasets demonstrated that the proposed model achieved state-of-the-art performance. Notably, these improvements were realized without significant increases in computational cost, underscoring the model’s efficiency and suitability for real-world deployment.

To further assess the model’s potential for real-world application, we conducted inference efficiency tests on a laptop equipped with an RTX4060 GPU. The experimental results show that for a single input image of size 640×640, the average inference time is 6.6 ms, with preprocessing and postprocessing times of 1.4 ms and 1.0 ms, respectively. The overall average processing latency is approximately 9.0 ms, corresponding to an inference frame rate of about 111 FPS, and the model weight size is 16.0 MB. These findings indicate that the proposed model is well-suited for real-time and lightweight deployment, providing strong support for use in edge devices, mobile terminals, and other practical scenarios.

Although this study provides a new direction for the advancement of road damage detection, several limitations remain. First, the model’s localization accuracy still has room for improvement when detecting damage types with complex geometries and significant aspect ratio variations, such as extremely thin and elongated cracks, suggesting that its adaptability to target bounding box shapes requires further enhancement. Second, the VFNM module exhibits a certain sensitivity to annotation density during training; if annotations are uneven or missing, the distribution of attention to critical regions may be adversely affected. Additionally, while the model achieves significant improvements on the HKPC dataset, the extent of performance gains on the more complex and diverse RDD2022 dataset is relatively limited, revealing shortcomings in generalization ability and adaptability to non-fixed viewpoint samples.

Future works will focus on several directions: (1) exploring multi-scale adaptive frameworks and more flexible object representation methods to enhance geometric adaptability for damage targets with large aspect ratio variations; (2) optimizing the noise modulation mechanism of the VFNM module to reduce reliance on annotation density and improve robustness in sparsely annotated scenarios; (3) integrating self-supervised and transfer learning strategies to further boost generalization and robustness on large-scale, complex datasets; (4) optimizing the deployment pipeline for typical low-power edge devices to promote real-world application of the model in road infrastructure monitoring.

## Figures and Tables

**Table 1:** Comparison of Parameter and FLOPs Overhead Introduced by Stacking CBAM or R-CBAM Modules Three Times in the YOLOv10s Backbone. The table summarizes the model size and computational cost for the original YOLOv10s, YOLOv10s with CBAM stacked three times, and YOLOv10s with R-CBAM stacked three times.

Model	Parameters (M)	FLOPs (GFLOPs)	Parameter Increase (%)	FLOPs Increase (%)
YOLOv10s	8.07	24.8	–	–
YOLOv10s + CBAM (×3)	8.12	24.8	0.6%	–
YOLOv10s + R-CBAM (×3)	8.24	26.2	2.1%	5.6%

**Table 2:** Distribution of sample numbers and proportions by road damage type in the HKPC dataset.

Road Damage Type	Number of Samples	Proportion of Samples
Pothole	7971	38.0%
Alligator cracks	2892	13.8%
Transverse cracks	3666	17.4%
Longitudinal cracks	6468	30.8%
Total	20,997	100%

**Table 3:** Distribution of sample numbers and proportions by road damage type in the RDD2022 dataset.

Road Damage Type	Number of Samples	Proportion of Samples
Longitudinal cracks (D00)	26,016	47.3%
Transverse cracks (D10)	11,830	21.5%
Alligator cracks (D20)	10,617	19.3%
Potholes (D40)	6544	11.9%
Total	55,007	100%

**Table 4:** Performance comparison of object detection models on the HKPC and RDD2022 datasets.

Model	mAP@0.5 (HKPC)	mAP@0.5 (RDD2022)	GFLOPs	Parameters (M)
Faster RCNN	0.601	0.607	251.4	60.5
RT-DETR-L	0.623	0.619	108.3	32.8
YOLOv5s	0.572	0.567	24.0	9.1
YOLOv8n	0.621	0.618	8.7	3.0
YOLOv8s	0.625	0.623	28.4	11.1
YOLOv8l	0.633	0.628	164.8	43.6
RDD-YOLO	0.635	0.625	255.3	65.9
YOLOv10s	0.656	0.637	24.8	8.1
YOLOv10l	0.659	0.638	120.3	25.8
YOLOv11s	0.668	0.641	21.6	9.4
YOLOv12s	0.665	0.640	21.5	9.3
**YOLO-ERCD (Ours)**	**0.677**	**0.641**	26.2	8.2

**Table 5:** Ablation study of YOLO-ERCD based on the YOLOv10s model.

YOLOv10s	R-CBAM	CAGC	VFNM	mAP@0.5
✓				0.656
✓	✓			0.665
✓		✓		0.662
✓			✓	0.665
✓		✓	✓	0.667
✓	✓	✓		0.668
✓	✓		✓	0.675
✓	✓	✓	✓	0.677

**Table 6:** Comparison of CBAM integration positions in YOLOv10s.

Position	mAP@0.5
No CBAM	0.656
CBAM in Backbone	0.661
CBAM in Neck	0.659

**Table 7:** Comparison of CBAM and R-CBAM stacking in YOLOv10s (backbone).

Number of Stacked Layers	1	2	3	4
CBAM (mAP@0.5)	0.661	0.653	0.622	0.6
R-CBAM (mAP@0.5)	0.662	0.664	0.665	0.665

**Table 8:** Comparison of gamma correction strategies (mAP@0.5).

Gamma Correction Strategy	mAP@0.5
No gamma correction	0.656
Random gamma correction	0.658
CAGC Module (Proposed)	0.662

**Table 9:** Comparison of VFNM and Gaussian noise augmentation (different noise levels).

Noise Level (σ)	5	10	15	20	25
Gaussian Noise Augmentation	0.659	0.658	0.586	N/A ^1^	N/A ^1^
VFNM Module	0.661	0.665	0.651	0.643	0.622

^1^ Gaussian noise augmentation failed to converge at these variances due to excessive noise disrupting training.

**Table 10:** Performance of the model across categories in the HKPC test set.

Class	Precision	Recall	AP@0.5	AP@0.5:0.95
Pothole	0.798	0.737	0.785	0.422
Alligator cracks	0.867	0.573	0.702	0.405
Transverse cracks	0.662	0.570	0.570	0.245
Longitudinal cracks	0.688	0.594	0.652	0.349
All (mean)	0.754	0.618	0.677	0.355

**Table 11:** Performance of the model across categories in the RDD2022 test set.

Class	Precision	Recall	AP@0.5	AP@0.5:0.95
Longitudinal cracks (D00)	0.634	0.507	0.568	0.329
Transverse cracks (D10)	0.623	0.482	0.546	0.284
Alligator cracks (D20)	0.682	0.627	0.681	0.370
Pothole (D40)	0.679	0.731	0.770	0.488
All (mean)	0.655	0.587	0.641	0.368

## Data Availability

The dataset used in this study is available upon request.
